# Exploring Reliable and Efficient Plasmonic Nanopatterning for Surface- and Tip-Enhanced Raman Spectroscopies

**DOI:** 10.3390/ijms242216164

**Published:** 2023-11-10

**Authors:** Antonio Sasso, Angela Capaccio, Giulia Rusciano

**Affiliations:** 1 Department of Physics “E. Pancini”, University of Naples “Federico II”, 80126 Naples, Italy; angela.capaccio@isa.cnr.it (A.C.); giulia.rusciano@unina.it (G.R.); 2 Institute of Food Sciences, URT-CNR Department of Biology, University of Naples “Federico II”, 80126 Naples, Italy

**Keywords:** Raman spectroscopy, surface- and tip-enhanced Raman scattering, biophotonics

## Abstract

Surface-enhanced Raman scattering (SERS) is of growing interest for a wide range of applications, especially for biomedical analysis, thanks to its sensitivity, specificity, and multiplexing capabilities. A crucial role for successful applications of SERS is played by the development of reproducible, efficient, and facile procedures for the fabrication of metal nanostructures (SERS substrates). Even more challenging is to extend the fabrication techniques of plasmonic nano-textures to atomic force microscope (AFM) probes to carry out tip-enhanced Raman spectroscopy (TERS) experiments, in which spatial resolution below the diffraction limit is added to the peculiarities of SERS. In this short review, we describe recent studies performed by our group during the last ten years in which novel nanofabrication techniques have been successfully applied to SERS and TERS experiments for studying bio-systems and molecular species of environmental interest.

## 1. Introduction

Living systems are highly complex systems, which makes them challenging for quantitative investigations. Approaches in current biology and biotechnology research are increasingly aimed at the identification and precise characterization of basic processes on the level of individual cells or even biomolecules, like proteins, amino acids, and lipids.

Biophotonics is an emerging interdisciplinary research area, born from the merging of biology and photonics. Its purpose is to generate and handle light (photons) to image, detect, and manipulate biological materials [1,2,3,4]. The great advantage of using light derives from its non-invasiveness and from the numerous effects arising from light–matter interactions which allow researchers to probe the properties of bio-systems over a large spatial scale, ranging from organs or tissues down to single biomolecules. Similarly, in the time domain, basic molecular mechanisms can be studied over time down to the femtosecond scale. Photonic-based approaches have recently allowed researchers to shed light on the peculiar function of single proteins, DNA, and other important molecules. In medicine, biophotonics has introduced new ways to image and analyze living microorganisms, and for the diagnosis and treatment of diseases [5].

Confocal laser-based fluorescence microscopy is one of the most popular tools for the optical imaging of biomaterials, thanks to its high sensitivity and facile preparation of samples (staining). Generally, fluorescence imaging is achieved by using the intrinsic fluorescence of certain proteins or, more frequently, by resorting to the use of fluorophores bound to target molecules. Fluorescence provides information about the average life of excited states, quantum efficiency, and the degree of depolarization. Such parameters generally change depending on the fluorophore environment (polarity, pH, temperature, etc.) and, therefore, can be used as probes for biological sensors. On the other hand, all fluorescence-based techniques present several shortcomings: for instance, the presence of fluorophores can interfere with the normal behavior of the biomolecule and, most importantly, the chemical selectivity of fluorescence spectra is quite poor. Further, phenomena such as photo-quenching and photo-bleaching, in general, limit the observation time and, hence, the signal-to-noise ratio of the measurements [6,7,8].

Over the past decades, Raman spectroscopy (RS) has attracted much attention for its ability to overcome many of the limitations of fluorescence spectroscopy. Since the pioneering studies of the Indian physicist C.V. Raman (1928) [9], the Raman effect has remained for many decades a poorly investigated phenomenon due to the objective experimental difficulties related to the weak signals originating from inelastic photons. Thanks to the development of laser sources and low noise detectors, RS has recently become a widely used technique for many studies in physics, chemistry, materials science, environmental science, and, especially life science [10,11,12,13,14,15,16].

RS allows the label-free chemical recognition of molecular species by identifying their vibrational signatures, i.e., without any pre-treatment. Furthermore, it assesses the high specificity of the chemical composition of a sample thanks to the inherent and unique vibrational structure of molecular bonds (chemical fingerprinting). Indeed, there are other vibrational spectroscopic techniques, such as absorption spectroscopy and Fourier-transform infrared (FTIR) spectroscopy. While RS makes use of laser sources and detectors operating mainly in the visible and near-IR spectral range, absorption spectroscopy is based on infrared radiation which, as is well known, is much more difficult to generate and detect. More importantly, vibrational modes can be IR-active but not Raman-active, and vice versa. This constitutes an important limit for IR spectroscopy in the study of biological systems characterized by a high concentration of water characterized by a high dipole moment.

Nevertheless, RS, compared to other optical spectroscopies, such as absorption or fluorescence microscopy, has a low cross section (σ_Raman_ ~ 10^−10^–10^−15^ σ_fluo_), being based on a scattering phenomenon rather than a resonant one. This results in a significant limit in the detection of analytes at low concentrations (typically below μ-molar range).

This drawback has been overcome thanks to the accidental discovery of Fleishmann [17] who observed an amplifying effect of the Raman signal for molecules (pyridine) in contact with rough metallic surfaces illuminated by laser radiation. The phenomenon, called surface-enhanced Raman spectroscopy (SERS), was later interpreted in terms of localized surface plasmon resonances (LSPRs). LSPRs are coherent and collective electron oscillations at the interface between two materials, exhibiting the positive and negative real part of dielectric functions, respectively (typically metal–dielectric interface). Under proper experimental conditions (optical frequency and structure size), a huge amplification of the local optical field can take place, especially in the inter-particle gaps (hot-spots), leading to a remarkable enhancement of the interaction strength between photons and molecules. This amplification has proved to have great potential for numerous phenomena, including fluorescence, Raman spectroscopy, heat generation, photocatalysis, nonlinear optics, solar energy conversion, and so on [18].

A crucial role is played by the manufacturing techniques leading to the realization of nanostructured metal substrates. As will be discussed later, this has attracted a huge growth of studies aimed at the creation of reproducible, simple to create, and highly efficient plasmonic nanostructures [19].

An even more innovative LSRP-based technique is represented by tip-enhanced Raman scattering (TERS) [20,21,22]. In TERS, the optical field amplification is concentrated at a sharp metallic tip that is irradiated with laser radiation [23]. When the tip approaches the surface of the sample, it provides a localized region of plasmonic enhancement; hence, it behaves like a real nano-antenna able to scan the surface of interest of a wide variety of samples, with sub-diffraction-limited imaging capabilities. TERS tips typically consist of metallic scanning tunneling microscope (STM) tips or, more frequently, modified atomic force microscope (AFM) tips.

In this brief review, we begin by discussing the basic principles of Raman spectroscopy and its amplified variants SERS and TERS. Afterward, we will briefly discuss the two main approaches, bottom-up and top-down, used for the fabrication of plasmonic metallic nanostructures, highlighting their strengths and weaknesses. In particular, we will discuss in some detail two recently developed nanofabrication techniques, belonging, respectively, to the two bottom-up and top-down families. For both techniques, we will discuss their advantages and disadvantages, showing a selection of their applications to the study of bio-systems.

## 2. Basic Principles of Raman Spectroscopy

As is well known, photons of frequency *ν_inc_* can be scattered by molecules elastically or inelastically: the former are assigned to Rayleigh scattering, while the shifted photons are associated with Raman scattering.

In the context of a quantum description, Rayleigh photons derive from the excitation of a virtual energy level followed by an instantaneous decay towards the same starting vibrational level (*ν_Ray_* = *ν_inc_*). Conversely, Raman photons (*ν_Raman_* ≠ *ν_inc_*) can arise from two different decay channels. If the molecule occupies the lowest vibrational energy level, the decay occurs towards the first excited vibrational level, and the emitted photons exhibit an energy lower than the incident photons (Stokes photons, *ν_Stokes_* < *ν_inc_*). On the other hand, when the molecule is initially on an excited vibrational level, the decay can take place towards the fundamental level and the emitted photons show an energy higher than the incident photons (anti-Stokes photons, *ν_anti-Stokes_* > *ν_inc_*). In both cases, the Raman signal involves a coupling to the internal degree of freedom of the molecule, i.e., the molecular vibration of frequency *ν_vib_*. In particular, Raman photons have frequency *ν_Raman_* = *ν_inc_* ± *ν_vib_*, where the sum/difference results in anti-Stokes/Stokes Raman scattering, respectively. According to Boltzmann’s statistics, at room temperature, the vibrational levels are poorly occupied; therefore, the anti-Stokes signal is much less intense than the Stokes one. Importantly, since the shift in energy of Stokes photons is associated with the discrete vibrational modes of polarizable molecules, Raman spectra represent an analytical tool for determining the biochemical composition of the investigated analyte (“chemical fingerprint”). In this regard, RS provides information quite similar to that deriving from IR absorption. However, the two techniques have experienced a quite different fortune for biological applications. This is mainly due to the strong IR-absorption cross section of water, which usually masks the contribution of species in aqueous environment. In contrast, water interferes only poorly with Raman spectrum of aqueous solutions, due to the quite low water Raman activity.

Focusing on the study of biological material, the most significant spectral regions fall within 400–2000 cm^−1^ wavenumbers, typically associated with the bond vibrations of relevant macromolecules. In particular, Amide I-III protein bands fall in this range and provide useful insight on protein secondary structures; carbohydrates lie in the 1500–1700 cm^−1^ spectral range, while phosphate groups of DNA can be found in the 470–1200 cm^−1^ region. Higher-frequency bond vibrations associated with CH, NH, and OH stretching in lipids and proteins are instead observed in the range 2700–3500 cm^−1^ [24].

However, due to the high number of vibrational degrees of freedom that occurs in complex molecules made up of numerous atoms, a typical Raman spectrum of biological material is overly complex, and the extraction of biological information is quite challenging. Therefore, multivariate analysis approaches are often necessary to distinguish the different molecular components present in the specimen.

The other side of the coin of Raman analysis is related to the exceptionally low probability of occurrence of Raman scattering: each Raman photon corresponds to ~10^6^ Rayleigh scattered photons. The strength of the Raman effect can also increase by some order of magnitude if the laser frequency is resonant with molecular electronic transitions (resonance Raman scattering, RRS), but in such conditions, the contrast of Raman peaks could be reduced by the co-presence of a broad fluorescence spectrum.

Such negative features of Raman spectroscopy have relegated this technique to specific and rare applications for decades after its discovery. Its recent impressive diffusion is to be found in the extraordinary technological progress related to efficient laser sources, low-noise solid-state detectors, and effective filters to remove Rayleigh background. From an experimental point of view, a Raman spectrum is recorded through the following steps: collecting the scattered photons, rejecting the intense Rayleigh photons (usually by a notch-filter), dispersing the inelastic photons by means of efficient diffraction gratings, and recording the peaks with low-noise CCD camera [25].

In recent times, Raman spectroscopy has been combined with confocal optical microscopy (micro-Raman spectroscopy) and even with optical tweezers (Raman tweezers) [26,27] to analyze single microparticles, bacteria, or cells [28,29].

The spatial resolution of a micro-Raman system is limited by the diffraction of light and, according to Abbe’s law, it is d_Abbe_ = λ/2NA, where λ is the used laser wavelength and NA is the numerical aperture of the objective lens. For visible radiation and high NA objectives, d_Abbe_ is ~0.300 μm in the transverse plane, while in the axial direction it is ~1.2 μm. It is clear that, for extended samples, it is possible to acquire the Raman spectra on a two- or three-dimensional array of points, with a step comparable with the spatial resolution (raster scan). The collected spectra can then be analyzed in terms of the intensity of a selected Raman band corresponding to a specific bio-chemical component of the sample. The result is a spatial image of the abundance of that specific component (Raman imaging). Nowadays, Raman imaging is becoming a tool for a patchwork of interdisciplinary research, involving physicists, chemists, biologists, and molecular biologists and clinicians.

Alongside the many advantages of RS, however, it should be emphasized that the cross section of Raman scattering is quite low, typically ~10^−25^−10^−30^ cm^2^, depending on whether the process is resonant or non-resonant. In a typical Raman measurement using a diffraction-limited laser beam, the typical detection volume is of the order of few fL and, for concentrations of ~1 μM, the total number of scattering molecules is ~10^6^. Even after optimizing the power of the Raman probe and the integration time, the obtained signal approaches a signal-to-noise ratio close to 1, i.e., clearly not enough for many applications in which the analytes are present at sub μM concentrations, or for very thin samples.

As will be discussed in the next paragraph, this drawback has been overcome thanks to the discovery of the SERS (surface-enhanced Raman scattering) technique which pushes the limit of detection even to the single molecule level [30].

## 3. Surface- and Tip-Enhanced Raman Scattering

The discovery of SERS dates backs to 1974, when Fleishmann and co-workers [17] accidentally observed an anomalous enhancement of the Raman signal when pyridine molecules were deposited over a roughened silver surface. A few years later, two independent papers [31,32] provided a physical interpretation of the phenomenon based on localized surface plasmon resonances (LSPRs) induced by the laser radiation shining on metal nanostructured surfaces.

Since this initial discovery, there has been an impressive growth in the number of publications that have confirmed this phenomenon, and the SERS technique is today a powerful spectroscopic tool with high sensitivity and chemical selectivity [33,34,35]. Research activity in the field of plasmon-enhanced spectroscopies and their numerous applications is nowadays rooted in many laboratories scattered around the world. An indicator of the great interest in this research topic is the huge number of publications: by searching various databases using the keyword SERS more than 3 × 10^4^ papers are found. Even in Italy, there is very lively activity on this topic, but the detailed description of these studies is beyond the scope of the present paper. Here, we limit ourselves to mentioning only a few recent and remarkable reviews and book chapters by Italian authors [36,37,38,39,40,41,42]. For more information on the trend of Italian research, one can visit the website www.plasmonica.it (accessed on 14 September 2023). Among the countless applications, we note the most significant ones: environmental science [43], cancer detection [44], free immunoassay platform [45], food safety [46], and, very recently, coronavirus detection [47].

Coming to the basic physical principles, LSPR originates from the interaction between laser radiation and free electrons, which causes coherent and collective electron oscillations at the interface of two media having the real part of dielectric functions of the opposite sign. This condition is usually realized at the interface between noble metals (mainly gold and silver) and dielectrics. Noble metals exhibit resonance wavelength located typically in the visible and near-infrared regions. Although metals are the most used plasmonic materials, significant plasmonic responses are also observed in heavily doped semiconductors and 2D materials.

The plasmon frequency is certainly the most representative parameter of plasmonic effect:

(1) $$\omega_{p}=\sqrt{\frac{n_{e}e^{2}}{m^{*}\epsilon_{0}}}$$

where $n_{e}$ is the electronic density, e is the elementary charge, $m^{*}$ is the effective electron mass, and $\epsilon_{0}$ is the vacuum dielectric constant. For many metals, $\omega_{p}$ lies typically in the UV region.

A full description of LSPR is based on Maxwell’s equations [48], but this is beyond the scope of this review. A simplified approach to describe SERS is based on a metal spherical nanoparticle (NP) illuminated by laser light. The oscillating electromagnetic (EM) field can sustain collective oscillating surface plasmonic multipoles. If the size of the NP is small compared to the wavelength of the incident light, the plasmonic wave packet remains confined around the metal surface, giving rise to a surface plasmon (SP). Moreover, if the frequency of the incident EM field is close to the SP oscillation frequency, the field extinction (i.e., the sum of absorption and scattering) presents a maximum. It is not difficult to image that the SP resonance depends critically on the size and shape of the employed NP. The plasmonic response of NPs has attracted considerable interest since ancient times, when they were used as decorative pigments in stained glass and artworks. Probably the most famous example of such an application is the Lycurgus Cup (4th century), which exhibits different colors depending on whether light is passing through it. As already said, a comprehensive model of SP resonances is based on Maxwell’s equations with appropriate boundary conditions. A useful approximation scheme is the so-called electrostatic approximation, in which the optical field is considered constant over distances comparable to particle size, so that phase delay effects can be neglected (in practice, for visible radiation, the electrostatic approximation works well for objects of typical sizes up to ~10 nm). However, fully analytical solutions of Maxwell’s equations exist in a few selected simple geometries (Mie theory), as spheres and cylinders, while for generic geometries, numerical simulations are used. For simple geometries, like a sphere, the particle behaves as an induced dipole whose polarizability depends on the dielectric constants as follows:

(2) $$\alpha =\varepsilon_{m}V\frac{\varepsilon -\varepsilon_{m}}{\epsilon +2\varepsilon_{m}}$$

where *V* is the particle volume, $\varepsilon =\varepsilon_{r}+i\varepsilon_{im}$ is the complex frequency-dependent dielectric function of the metal, and *ɛ_m_* is the dielectric constant of the surrounding medium.

**Figure 1:**
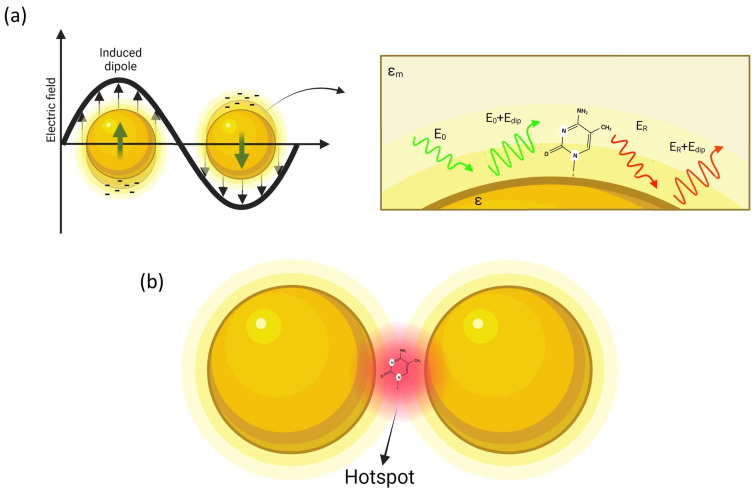
Simple description of SERS: (**a**) a metal nanoparticle illuminated by light causes the oscillation of the free electrons of the surface, so that a molecule placed at nanometric distance from the NP surface is affected by an amplified optical field; (**b**) amplification is further increased in the gap region in a dimer structure (hot-spot).

As depicted in Figure 1, a molecule in proximity of the sphere (at distance *d*) is exposed to a field *E_tot_*, which is the superposition of the incident field *E*_0_ and the dipole-field *E_dip_*:

(3) $$E_{tot}=E_{0}+E_{dip}=E_{0}+r^{3}\frac{\varepsilon -\varepsilon_{m}}{\varepsilon +2\varepsilon_{m}}E_{0}\frac{1}{{\left(r+d\right)}^{3}}$$

It is possible to define an optical gain as the ratio of the total field *E_tot_* at the molecule position to the incoming field *E*_0_:

(4) $$G\left(\nu_{L}\right)=\frac{E_{tot}}{E_{0}}∼\frac{\varepsilon -\varepsilon_{m}}{\varepsilon +2\varepsilon_{m}}E_{0}{\left(\frac{r}{r+d}\right)}^{3}$$

Despite the simplified model used, Equation (4) shows two notable results. Firstly, the denominator leads to a strong resonance when, at the laser frequency $\nu_{L}$, $\varepsilon +2\varepsilon_{m}$ reaches a minimum. The second interesting aspect is the dependence on the third power of the distance *d* between the analyte and the metal surface.

A second amplification step occurs because the Raman field itself is also enhanced. Therefore, considering both the incident and Raman field amplification, the total electromagnetic gain *G* of the Raman signal intensity is

(5) $$G_{tot}={\left|A_{inc}\right|}^{2}{\left|A_{Raman}\right|}^{2}∼E_{0}^{4}{\left|\frac{\varepsilon \left(\nu_{L}\right)-\varepsilon_{m}}{\varepsilon \left(\nu_{L}\right)+2\varepsilon_{m}}\right|}^{2}{\left|\frac{\varepsilon \left(\nu_{R}\right)-\varepsilon_{m}}{\varepsilon \left(\nu_{R}\right)+2\varepsilon_{m}}\right|}^{2}{\left(\frac{r}{r+d}\right)}^{12}$$

where $\nu_{L}$ and $\nu_{R}$ are the frequencies of the incident field and Raman photons, respectively. Interestingly, Equation (5) shows that the gain factor *G* decreases as (1/*d*)^12^, which is responsible for the local character of SERS enhancement [49].

In a real measurement, to give an estimation of how much the *SERS* signal (*I_SERS_*) is greater than the spontaneous Raman (*SR*) signal (*I_SR_*), the two signals have to be properly normalized. This is commonly introduced as the enhancement factor *EF*, defined as follows:

(6) $$EF=\frac{I_{SERS}}{I_{SR}}\frac{N_{SR}}{N_{SERS}}\frac{P_{SR}}{P_{SERS}}\frac{\tau_{SR}}{\tau_{SERS}}$$

in which the intensities *I_SERS_* and *I_SR_* are normalized to the respective number of detected molecules, and to the laser power and integration time employed in the respective measurements.

It is worth noticing that, in the electrostatic approximation, the plasmon resonance does not depend on the size of the NP. Nevertheless, following a more rigorous approach (Maxwell’s equations), it is possible to show that, considering the boundary conditions of the optical field, the size of the NP plays a significant role. As a general rule, as the NP size increases, (i) the resonances shift to the red, (ii) they are strongly damped and spectrally broadened, and (iii) new resonances appear because of the activation of multipolar resonances (such as quadrupolar resonances).

Although the single particle model is effective for understanding the basic mechanism of plasmon amplification, the intensity of the optical gain is not particularly high. This is the reason leading to the use of more complex nanostructures. The next step is to consider, for example, two nanospheres placed at a nanometer distance. It is possible to demonstrate that resonance coupling produces a red shift of the plasmon resonance, and, still more interestingly, the field amplification is mainly focused on the gap separating the two spheres, as illustrated in Figure 1b. These highly localized regions of intense local field enhancement are also observed within the interstitial crevices present in metallic nanostructures, and are called “hot-spots” [50]. Such hot-spots provide huge enhancements of up to 10^14^ orders of magnitude in areas of subwavelength localization, allowing to reach sensitivity at single molecule level [51].

For gap distances below ~1 nm, the classic electromagnetic theory is no longer valid, and a quantum mechanical approach is necessary. Interestingly, as a rule, the SERS signal shows a critical dependence on the distance from the hot-spot because it drops to zero within a few tens of nm. As we will see later, this peculiarity plays a key role in the study of interfaces.

The enhancement effect just described is commonly referred to as electromagnetic enhancement. However, another cause of SERS enhancement is the so-called chemical enhancement due to electron transfer between the modified molecules and nanoparticles [52]. In general, chemical enhancement contributes to SERS enhancement by a minor factor, of about 10 ÷ 10^3^.

Even SERS, like any experimental technique, has its limits. One of these is certainly represented by the large fluctuations of the SERS signal, which make reproducible experiments exceedingly difficult. The cause of such fluctuations is primarily the heterogeneity of the SERS substrate which leads to a variable hot-spot density when moving from point-to-point on the substrate surface. Another cause is the position and orientation with which the analyte occupies the hot-spot sites, and even the intermittent contact with the substrate.

This problem becomes particularly important when the number of molecules in the laser spot is greatly reduced, reaching the single molecule regime. In ref. [53], the authors discuss the phenomenology and statistics of this kind of fluctuation.

The variability of the SERS signal both over time (repeated measurements at the same point) and over space (repeated measurements at different points) has prompted an effort, still in progress, to fabricate SERS substrates as reproducible as possible. Nevertheless, since this aspect cannot be completely eliminated, it is necessary to resort to multivariate statistical techniques, among which principal component analysis (PCA) is the one most widely used. This aspect is particularly significant when complex biological structures are investigated, due to the intrinsic heterogeneity of this type of sample.

Although the hot-spots are extremely localized regions, their position within the area of the focused laser beam remains undetermined; therefore, the SERS technique remains a diffraction-limited technique. In other words, if there was only one molecule in the laser area, we would not be able to say from which hot-spot the SERS signal originates.

This drawback has been solved by a recent technique named tip-enhanced Raman spectroscopy (TERS) [54,55,56,57,58]. Tip-enhanced Raman spectroscopy is a combination of the plasmonic amplification, typical of LSPR, with scanning probe microscopy (SPM), such as atomic force microscopy (AFM), or similar microscopies such as scanning-tunneling microscopy (STM). The general idea is to use solid metal tips or dielectric tips coated with nanostructured metal films. The enhancement mechanism is strongly dependent on the geometry and the type of plasmonic material of the probe. By modifying these parameters, it is possible to manipulate the conditions necessary to excite the plasmon resonance modes in the tip apex that contribute to the local field enhancement. When the TERS tip approaches the surface of the sample under investigation and is illuminated by laser radiation, it behaves as a nano-antenna [59,60,61,62,63], and a localized nanoscale enhancement of the Raman signals is produced from the portion of the sample placed under the tip [64,65].

The near field at the tip apex results significantly amplified for longitudinally polarized far field excitation [66]. Moreover, when the tip is close to a metallized surface, a much higher gain is achieved in the gap between the tip and the substrate. This configuration, named gap-mode TERS, leads to a further enhancement of ~10^2^, which is very useful for most biomolecular applications [67,68].

TERS spatial resolution depends on the radius of curvature of the tip, typically varying around 10–20 nm [69]. However, single-molecule mapping has been published with a higher spatial resolution of 0.5 nm [70].

In this work, we will not go into detail on the numerous technical aspects of the TERS technique (illuminating geometries, the role of the polarization, and so on). For this purpose, we refer to several exhaustive review articles [22,71,72,73,74,75,76].

TERS amplification is usually described in terms of the so-called optical contrast (OC), defined as the ratio of near-field (*I_NF_*) to far field (*I_FF_*) Raman intensities:

(7) $$OC=\frac{I_{NF}}{I_{FF}}=\frac{I_{tip}-I_{0}}{I_{tip}}$$

A high contrast value implies a strong plasmonic TERS enhancement at the apex of the tip. The intensity *I_tip_* is the TERS experimentally detected Raman intensity which is superimposed on a large non-enhanced background *I*_0_. In terms of OC, the *EF* can be approximated by the relation:

(8) $$EF\approx OC\frac{A_{FF}}{A_{NF}}$$

where *A_FF_* = *πw*^2^, with *w* being the beam waist, and *A_NN_* ~ *πr*^2^, with *r* being the tip radius of curvature.

Finally, a common issue which concerns both SERS substrates and TERS tips is their lifetime [72,77]. The exposure of these devices to the air or other environments can induce several types of mechanisms (mainly oxidation and contamination by the adsorption of molecules present in the environment) which reduce plasmonic activity and/or produce significant interferences in the final spectra. Thus, many strategies have been proposed both to fabricate high-quality TERS probes and to extend the tip’s lifetime. This feature will be briefly addressed in the next sections [78].

## 4. Development of Plasmonic Platforms

For the development of high-performance plasmonic platforms, it is equally important to provide strong signal enhancement and reproducibility. Insufficient reproducibility has long been a major obstacle to the use of this analytical method in critical sensing applications [79].

**Figure 2:**
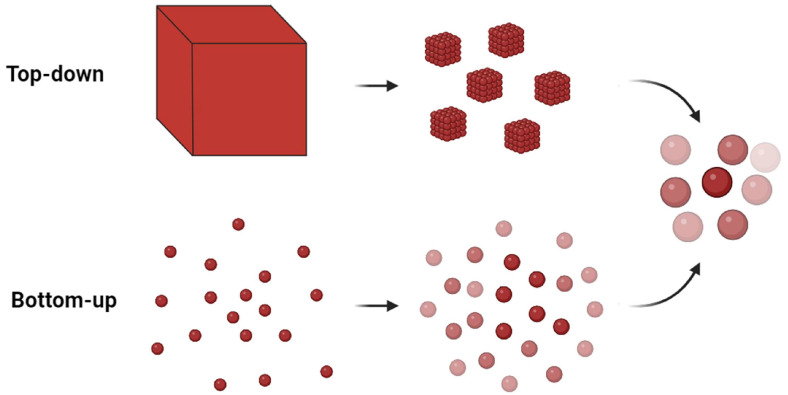
Schematic representation of the bottom-up and top-down approaches.

This explains why, in recent decades, a great effort has been made to create metallic nanopatterns exhibiting the following main properties: efficiency, reproducibility, ease of production, and low costs [19,80,81,82]. All these methods can be subdivided into two main categories: bottom-up and top-down (see Figure 2) [83,84].

In bottom-up techniques, one starts from individual atoms to aggregate them and gradually arrive at nanoscale structures. Vice versa, top-down techniques deal with dividing or structuring a macroscopic piece of material in small parts.

Both approaches present some inherent problems, with specific advantages and disadvantages, so that the selection of the appropriate fabrication procedure depends on the nature of the specific application. The advantages and disadvantages of these two approaches will be briefly discussed in the following. Later in the text, we will focus on two techniques developed in our laboratory and successfully applied to the study of different bio-systems. The first technique belongs to the group of bottom-up approaches, and is based on the ability of certain systems to self-organize through chemical interactions to give rise to nanometric structures which have a high degree of order, comparable to that obtained by nanolithography techniques. The second technique, belonging to the group of top-down approaches, is based on the solid-state dewetting (SSD) of a thin Ag layer able to produce a SERS-active substrate characterized by a degree of porosity (coral-like).

### 4.1. Bottom-Up Approaches

Typical bottom-up approaches rely on traditional methods for synthesizing metal NPs, introduced for the first time by Lee-Meisel [85] and Creighton [86]. These techniques have significantly evolved, to the point of producing an extraordinary variety of NPs, including core–shell NPs, multi-shell NPs, and so on. The final size and shape of the synthesized NPs is determined in a sufficient way by the amount of additives, the concentration of reactants, and the preparation conditions [87,88]. Nanoparticle aggregation is an important issue, since the highest SERS enhancement factors are expected in the nanosized gap between two close metallic NPs. The aggregation of the NPs in aqueous suspensions (colloids) can be controlled either by steric constraints or by electrostatic repulsion. Unfortunately, the colloids, in some cases, show specific Raman modes assignable to citrate (reducing agent) or nitrate. Thus, the detection of the molecules of interest might be hindered by the strong SERS contribution of the aggregated metal colloids themselves [89].

Another family of bottom-up approaches, identified as “non-traditional”, is based on the Ag NP synthesis occurring through high-temperature reduction in porous solid matrices [90,91], vapor-phase condensation of a metal onto a solid support [92,93,94], laser ablation of a metal target into a suspending liquid [95], photoreduction of Ag ions [96,97], and electrolysis of an Ag salt solution [98,99].

Characteristic advantages of bottom-up approaches are the following: (i) no specialized equipment is necessary; (ii) solution-based processing and assembly can be readily implemented; and (iii) large quantities of NPs can be synthesized [100].

However, these advantages are countered by some limitations. For instance, considering traditional methods, the major problem is often a limited flexibility in the size of the particles that can be produced (about 10 nm), while for optical applications, larger particles (around 80–100 nm) are often necessary.

Similarly, the major problems for the non-traditional methods often concern the following: (i) a wide size distribution, (ii) a lack of particle crystallinity, and (iii) the cost and scalability of the production.

Thus, the optimum synthetic method should address all the above problems, and additionally yield particles with no extraneous chemicals that can potentially alter the particle’s optical properties and surface chemistry. The above discussion certainly does not provide a complete list of the available synthetic methods, but most likely a variant of a broad representation of what has been reported. The use of colloidal SERS is widespread as the substrates are easy to synthesize at low cost and are also highly dispersible [101]. Nevertheless, colloidal-based substrates are limited in routine trace analysis due to the ‘basic reproducibility criteria’ which have not been properly addressed [102].

### 4.2. Top-Down Approaches

Top-down approaches are mainly based on photolithography or particle beam lithography. In photolithography, after the patterning of the poly methyl methacrylate (PMMA) resist and the subsequent reactive ion etching, metal is deposited over the patterned structure.

Due to the free individual selection of the lateral shape and the height of the NPs, the LSPR of the generated NPs can be easily tuned from the IR to the near-UV. This flexibility is the prerequisite for several fundamental and practical applications. The major obvious advantages of the synthesis of photolithography are the highly ordered metal nano-textures with a narrow shape and size distribution on substrates. Photolithography is also a reliable process, which is essential for mass production. As to its drawbacks, photolithography is diffraction-limited.

Nanosphere lithography (NSL), invented in the 1980s by U. Fischer [103], in contrast to the above-mentioned techniques, is inexpensive, allowing a high throughput. This technique provides highly ordered NPs with lateral sizes between 30 nm and several micrometers. The principle is the deposition of the nanospheres from a solution onto a carefully cleaned substrate (using spin-coating, tilt-coating, or dip-coating). Thus, a monolayer of hexagonally ordered nanospheres on the substrates is obtained by self-assembly, acting as the lithographic mask during the subsequent deposition of the metal. Once the nanospheres are removed, they leave a highly ordered array of triangular NPs. Nevertheless, NSL is constrained to triangular- or hexagonal-shaped NPs arranged in a defined order on the substrate.

Electron-beam lithography (EBL) is a direct drawing technique and differs significantly from photolithography. Theoretically, nanostructures with lateral dimensions below 1 nm are possible due to the short de Broglie wavelength of electrons [104], hence, well below the diffraction limit. More realistically, the resolution is limited to approximately 10 nm because of the proximity effect, that is, the forward and backward scattering of the electrons in the resist. As for its drawbacks, it is a time-consuming serial process and requires clean rooms and special nanofabrication tools and, hence, quite expensive [105].

### 4.3. TERS Probe Production

Most of the techniques described above for the production of SERS substrates were also used to decorate AFM tips with metallic nanostructures, in order to transform them into probes for the TERS technique. While scanning tunneling microscopy tips used for TERS typically consist of etched bulky silver or gold probes with a sharp apex, AFM TERS tips are typically fabricated via vapor depositions of metal coatings on commercial silicon probes [106,107,108]. Vapor deposition typically produces a monolayer of NPs resulting from the dewetting of the metal on the AFM surface, which produces rough films or discontinuous random nano-islands [109]. Other methods have been proposed such as isolated NP deposition [54,110], and electrochemical [111] or pulsed electrodeposition [112].

## 5. Silver-Based Nanopatterns Obtained by Self-Assembling of Block Copolymers

Block copolymers are defined as macromolecules containing two or more polymer chains that are bound together through covalent bonds [113,114,115]. The simplest block copolymer is the diblock copolymer, which consists of two different polymer chains covalently attached at their ends. They undergo segregation into a variety of ordered structures due to the repulsion of the immiscible blocks, much as in the case of a blend of immiscible homopolymers. The length of the polymer chain determines the length scale of microdomains.

Block copolymer thin films are of particular interest due to the possibility of obtaining two-dimensional patterns with very high registry and regularity. As a matter of fact, they have received significant attention over the last few decades because of their ability to self-assemble in the nanometer length scale [115,116]. This self-assembly is caused by both the incompatibility of the two polymer blocks and their covalent connectivity. The ability of soft materials such as block copolymers to form a rich variety of nanoscale periodic patterns offers the potential to fabricate high-density arrays for use in different applications [113,117,118]. The formed self-assembled patterns are considered both as nanolithography masks and templates for the further synthesis of inorganic or organic structures. All these appliances depend on the extremely regular self-assembly of block copolymers over macroscopic distances. More generally, self-assembly techniques are attractive because of their highly parallel nature and enabling of large-scale patterning rapidly and at very low costs. The flexibility of the technique extends its use from highly ordered nanostructures over large surface areas [117,119] to the generation of metal NPs [120] and structured metal surfaces [121,122].

Nowadays, the block-polymer-based approach presents a powerful route to the “bottom-up” fabrication of nanostructures because of the block copolymer’s ability to self-assemble in the nanometer length scale [115,118] caused by both the incompatibility of the two polymer blocks and their covalent connectivity.

### 5.1. Fabrication of SERS Substrates and TERS Tips by Self-Assembling Block Copolymers

**Figure 3:**
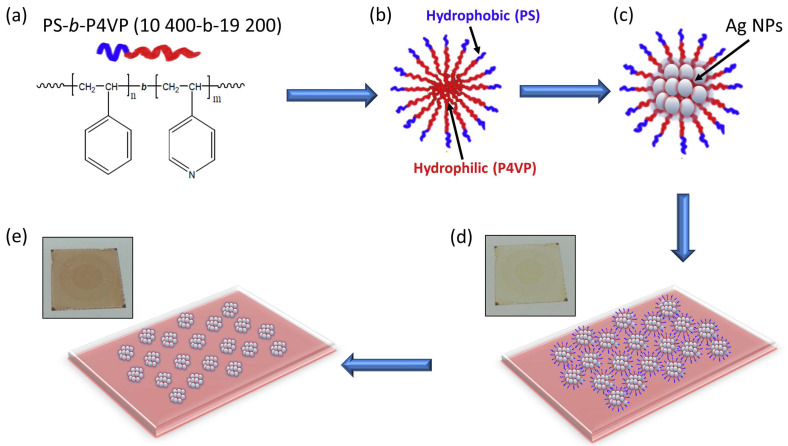
General scheme for the fabrication of the plasmonic template, according to the steps described in the text.

Given the above, let us describe our fabrication method of hexagonal periodic templates of silver NP clusters based on the immediate long-range self-assembling of block-copolymer (BCP) micelles [123]. In our case, we used as block copolymer polystyrene-block-poly-4-vinylpyridine (PS-b-P4VP) loaded with silver nanoparticles (Ag-NPs). The synthesis of the substrates consists of the following steps (see Figure 3):

(a) BCP selection;
(b) Formation of the PS-b-P4VP crew-cut micelles in toluene solution;
(c) Incorporation of the silver precursor (silver ions) into the P4VP micelle core;
(d) Reduction by BaBH_4_ of the coordinated silver ions to silver NPs;
(e) Centrifugation and phase separation of the solution;
(f) Spin-coating of the solution on the glass substrate;
(g) Polymer removal by UV-light exposure.

**Figure 4:**
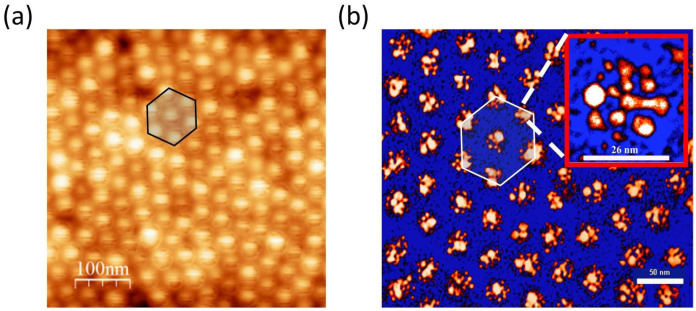
(**a**) Example of AFM morphological characterization of the micelle film on the glass substrate; (**b**) TEM micrograph of the plasmonic hexagonal lattice coated on glass and transferred on TEM carbon grid. Adapted from [123].

The packing of the BCP micelles was imposed by choosing a proper relative chain length of the block copolymers of PS and P4VP, defining the micelle’s shell and core, respectively (PS-b-P4VP of molecular weight 10400-b-19200). A typical AFM image of the resulting micelle distribution and a transmission electron microscopy (TEM) image of the Ag-BCP nano-patterned film are shown in Figure 4. The major results obtained are the uniform hexagonally ordered silver nanoclusters. Each nano-island had an average diameter D ~26 nm and height h ~14 nm, and consisted of nearly touching nanoparticles of size d in the range ~1–12 nm (see inset of Figure 4b). The hexagonal pattern was characterized by a gap g ~15–20 nm.

This gap could be reduced to 2 nm by increasing the polymer concentration and spin-coating speed, thus obtaining a higher density packing which exhibits near-hyperuniform long-range correlations [124].

The large-area characterization of the SERS intensity response over the random nanostructure was performed using crystal violet (CV) as probe molecules. This CV characterization led to an average enhancement factor EF = 1.04 × 10^6^. A relative standard deviation of 3.6% was found after sampling the SERS signal on a large area of 1 cm^2^. It is clear that this is a prerequisite to obtain reliable information from sample scans.

### 5.2. Study of Cell Membranes Using SERS

The plasma membrane defines the border between the interior of the cell and the outside environment. It plays a key role for many fundamental cell functions, such as cell signaling and trafficking, thanks to the presence of a large variety of membrane proteins. When a cell is adhered to a SERS substrate, two types of Raman signal are created. The first contribution comes from the whole volume of the diffraction-limited Raman probe, and it is generated from a spontaneous Raman signal. The other contribution, instead, arises from the membrane portion adhered to the plasmonic nanostructure which is the one that had undergone SERS amplification. If the EF of the SERS substrate is adequate (of the order of 10^5^ or higher), the contribution from the spontaneous Raman signal can be considered negligible. In the following, we briefly discuss two examples concerning the study of cell membranes: red blood cell membrane and tumoral cell membrane.

**Figure 5:**
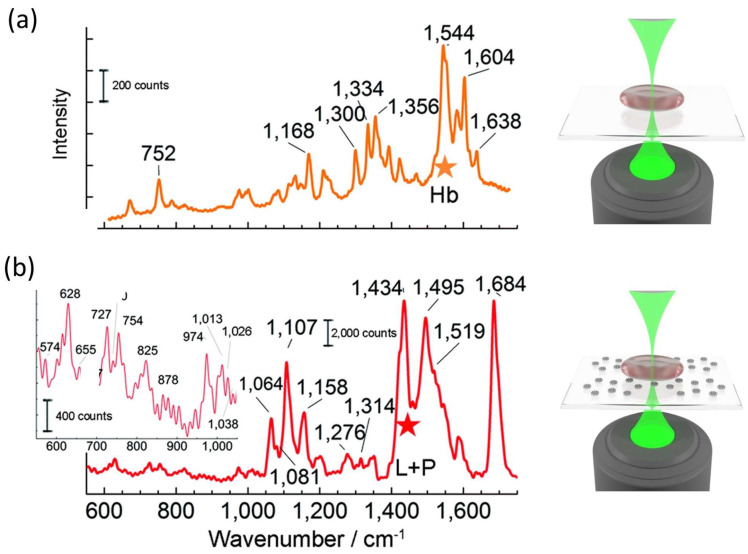
Comparison between typical signals in the case of RBCs adhered to a glass coverslip (**a**) and a SERS substrate (**b**). In both cases, a cartoon of the experimental configuration is provided on the right. Adapted from [123].

To highlight this potential, we examined red blood cells adhered to the SERS substrate. This is quite a challenging task, due to the occurrence of RRS for hemoglobin (Hb) present in the inner volume of the cell, using an excitation probe of λ = 532 nm. Figure 5 compares typical spectra obtained for red blood cells (RBCs) adhered to a glass coverslip (a) and to a SERS substrate (b). The excitation power for the SERS experiment was P_in_ = 3 μW and the integration time Δt = 100 ms, while Raman signals were acquired by using P_in_ = 500 μW and Δt = 1 s.

As expected, the spontaneous Raman spectrum was mainly ascribable to hemoglobin, with typical spectral features due to the heme-group. In contrast, the surface SERS spectra had a clearly different Raman fingerprint characterized by membrane peaks.

### 5.3. Study of Protein Overexpression in Cancer Cells

Membrane proteins play a crucial role in cell life, regulating fundamental processes such as ion exchange and interaction with their environment through receptors. Intriguingly, some proteins expressed in healthy cells are overexpressed in cancer cells. In this case, membrane analysis using SERS can constitute a valuable tool for the early diagnosis of malignant transformations of cells. Herein, we discuss the case of a model protein, carbonic anhydrase IX (CA IX) [125], in a tumor cell line (SKOV3). CA IX is a trans-membrane protein overexpressed by cancer cells as an expedient to neutralize the acidic pH resulting from the anaerobic metabolism. To prove the suitability of SERS to detect CA IX protein expression on the cell surface, SKOV3 cells were transfected with CA IX in the presence of a vector encoding nuclear enhanced green fluorescent protein (nEGFP), a fluorescent protein which allows to distinguish successfully transfected cells from untransfected ones. Doubly transfected SKOV3 cells, i.e., transfected with both CA-IX and nEGFP, are here indicated as CAIX+, while SKOV3 cells transfected with only nEGFP are indicated as CAIX−, and used as control.

**Figure 6:**
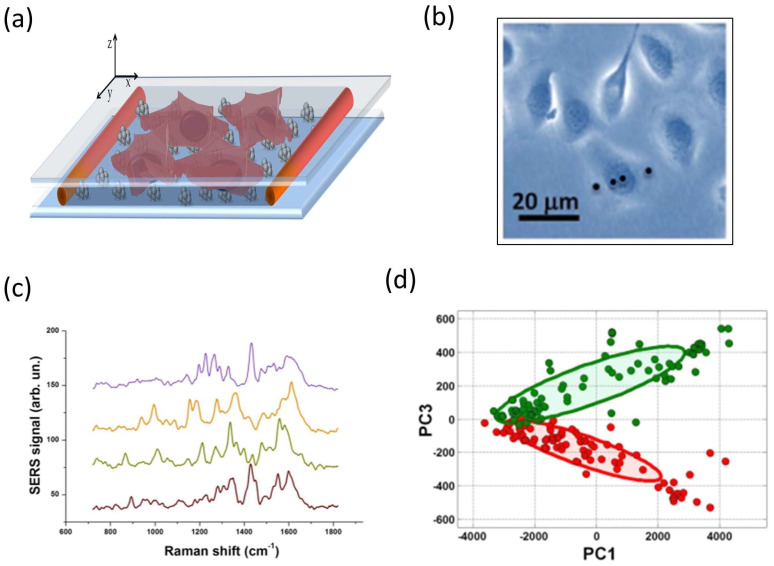
(**a**) Sketch of the sample cell used for SERS analysis, formed by a sandwich structure consisting in a nano-patterned Ag-substrate covered by a glass coverslip on which SKOV3 cells were cultured. (**b**) Bright field image from SKOV3 cells. (**c**) Four background subtracted SERS spectra acquired in the single cell shown in (**d**). Measurements were acquired with a laser power of 10 μw (on the sample) and an integration time of 2 s. (**d**) Two-dimensional scatter plot (PC1-PC3 plane). The 95% confidence ellipses relative to CAIX+ (red) and CAIX− (green) are also shown. Adapted with permission from [125], under the Creative Commons 3.0 license (https://creativecommons.org/licenses/by/3.0/ accessed on 8 September 2023).

Cells cultured on a glass coverslip were put in contact with the SERS substrate by simply laying down the cell coverslip onto the plasmonic substrate, allowing cell adhesion by gravity (Figure 6a). It is clear that, due to the great heterogeneity of the structure of a cell, there is considerable variability in the spectra as the area hit by the laser beam varies. For example, in Figure 6b, the SERS spectra acquired at the four points indicated in Figure 6c are compared. Therefore, to obtain a significantly representative spectrum of a cell, it was necessary to acquire spectra at numerous sites of the cell (N = 20) and calculate the average spectrum. It must also be said that averaged spectra of transfected and non-transfected cells do not show obvious differences, and this required an analysis based on principal component analysis (PCA). The outcomes of this analysis (Figure 6d) show quite a good separation between CAIX+ cells (red symbols) and CAIX− cells (green symbols). Applying the leave-one-out cross validation (LOOCV) procedure to test the robustness of our SERS-based assay of cellular membranes, we estimated a sensitivity of ~94%, a specificity of ~93% and a global accuracy of ~94% in our SERS-based approach for CAIX detection.

### 5.4. TERS Tip Fabrication and Characterization

**Figure 7:**
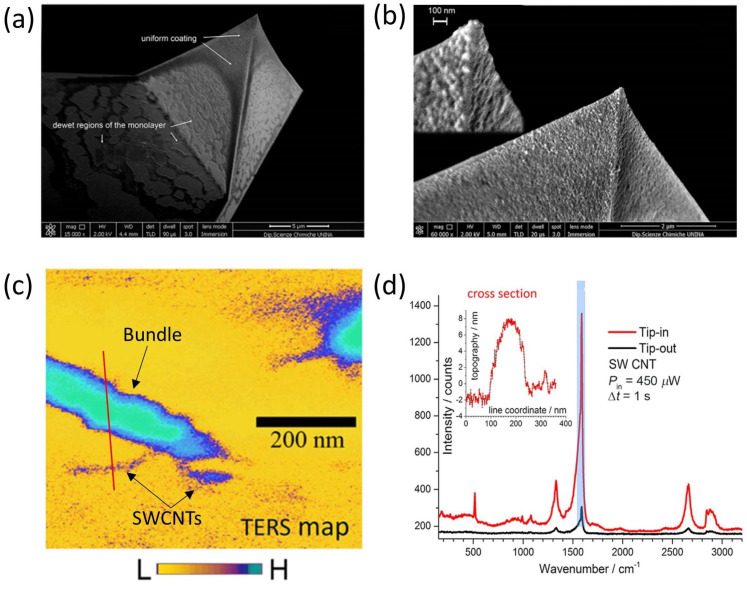
(**a**) SEM micrograph of an Arrow©-type AFM probe coated with clusters of AgNPs, showing a uniform monolayer on the pyramidal tip with dewetted regions at the base. (**b**) Magnified version of (**a**), with inset showing a region close to the apex where contrast is increased to resolve the structure of close-packed nano-islands. (**c**) Spatial distribution of TERS signal intensity relative to the Raman band at 1558 cm^−1^, acquired in a large area of bundles of SWCNTS spin-coated on glass. (**d**) Tip-in and tip-out TERS signals acquired on the bundle of SWCNTS. The band at 1588 cm^−1^ is highlighted in blue, and the inset shows the topographic cross section along the red line drawn in (**c**). (Adapted with permission from [126], under the Creative Commons 3.0 license (https://creativecommons.org/licenses/by/3.0/ accessed on 8 September 2023).

The self-assembling capability of block copolymers was also used to functionalize near-field scanning probes [126]. The coating of AFM scanning probes was performed using BCP micelles loaded either with clusters of silver nanoparticles (AgNPs) (as described in the previous section) or with a variant consisting of bimetallic structures of mixed species of silver and gold nanoparticles. The tip fabrication was carried out using dip-coating: for this process, it was crucial to extract the tip from the liquid with the apex pointing toward the liquid/air interface. This operation produced a monolayer of AgNPs-loaded micelles on the tip, constituting a sort of nanometal skin. Figure 7a,b present the SEM micrographs of Si-AFM (Arrow©-type probes—NanoWorld) coated with metal nano-islands. To determine the spatial resolution and the optical contrast (OC) of the TERS probes, a sample of single-walled carbon nanotubes (SWCNTs) spin-coated on a glass coverslip was used. For these experiments, TESPA tips (Bruker), having a nominal radius of curvature of 8 nm, were employed as supporting AFM probes coated with Ag clusters. Figure 7c shows the TERS signal distribution of the characteristic Raman band at 1588 cm^−1^ of the SWCNT spectrum. The comparison of the typical TERS signals when the tip is near and far from the SWCNT sample is shown in Figure 7d. By taking into account these results, we were able to estimate an OC of ~35 and an EF of ~2.7 × 10^5^ for the tested TERS probes. In addition, a spatial resolution of ≃15 nm was found, considering the topographic cross section along the red line drawn in Figure 7c (see inset of Figure 7d).

## 6. Nanostructured Ag Films using Solid-State Dewetting

A second approach to fabricate nanostructured metal films developed by our group is based on a general mechanism inspired by solid-state dewetting (SSD). SSD is a phenomenon in which a thin metal film tends to agglomerate in small islands of nanometric dimensions when subjected to external perturbations, such as heating. Although in some cases, where the continuity and integrity of thin films are required, this mechanism is undesired, in recent years, SSD has emerged as a novel, simple, and low-cost method for the fabrication of NP patterns [127]. SSD has been employed in various fields (catalysts, resistors, electrodes, and sensors [128]), and has recently attracted interest in the field of plasmonic devices due to the easy tuning of the optical resonances by acting on the process parameters [129,130,131].

The physical mechanism governing SSD is the minimization of the total surface energy of the free surfaces of the film and substrate, and of the film–substrate interface. A thin metallic layer deposited on a substrate is in an unstable or metastable state, because of the relatively high energies of the metal surface. This no-equilibrium condition causes the metal layer to break up and agglomerate (dewet) in nano-islands, even at temperatures well below the metal’s melting point [128].

We have recently successfully implemented a new method based on the SSD mechanism to prepare nanoporous silver-based plasmonic substrates. The fabrication occurs following the application of a radiofrequency (RF) discharge (cold plasma) in synthetic air atmosphere, where the oxidation of the silver layer plays a significant role.

The mechanism is complex and not yet fully understood. In addition, some other mechanisms could play a role:

(i) The reactive species (atomic oxygen) produced in the discharge can oxidize the silver layer [132]. As the oxide layer grows, internal stresses due to mechanical or thermal excursions can induce the cracking of the formed layer [133]. However, as experimentally observed by the same authors and by our group, this cracking-induced process is not sufficient to explain the growth of nanostructures on the silver surface.
(ii) Another mechanism is oxide diffusion at the interface (Kirkendall effect [134]). The different diffusion rates of metals and oxides produces a disequilibrium of the material flow that is compensated with the inter-diffusion of vacancies; the condensation of excess vacancies can give rise to void formation (Kirkendall voids), which leads to the formation of nanopores [135]. Nevertheless, since the Kirkendall effect cannot be activated unless the material is exposed to an elevated temperature, the authors of ref. [136] suggest that the temperature could rise locally due to the exothermic nature of the chemical oxidation reaction of silver. Another explanation could be that the energy carried by the oxygen atoms is converted into heat in the oxide layer [133]. Nevertheless, the Kirkendall effect is considered responsible for the formation of nanovoids inside nanocrystals and below the formed oxide layer. In fact, this effect is used to produce hollow structures, as reported in several works [137,138,139].
(iii) Compared to the works of El Mel’s group, we suggest that the formation of porous nanostructures could be triggered by the solid-state dewetting of the oxidized silver layer. To minimize the energy of the system as the exposure time to the RF plasma increases, the structure of the oxide film rearranges to form grain boundaries along which atoms can easily migrate to form islands. In fact, a similar process is also reported in the work of Zhao et al. [140], where the Kirkendall voids have been proposed to explain the initial stage of thermally induced SSD of Cu films deposited on native Si oxide substrates. Our assumption is that the cracking process and the Kirkendall effect might weaken and destabilize the thin oxide layer, promoting the SSD of the silver oxide. Moreover, the morphology of the silver layer is more like the classical picture of spinodal dewetting because the nanostructures exhibit similar spatial correlation.

### 6.1. Fabrication of SERS Substrates and TERS Probes

Differently from the method based on self-assembling block copolymers, the procedure for the fabrication of SERS and TERS probes based on SSD is essentially the same. Here, we focus on the TERS tips.

**Figure 8:**
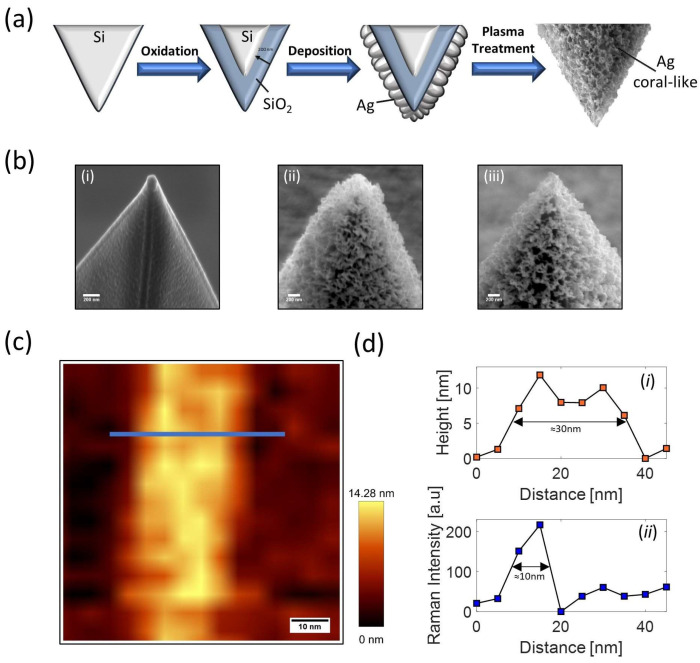
(**a**) Schematics of the procedure designed to fabricate Ag coral-like probes. (**b**) SEM images of a treated TESPA tip: (i) probe after sputtering of a 30 nm thick Ag layer; (ii) Ag-coated tip after air-based plasma treatment; (iii) TERS probe after Ar plasma reduction. (**c**) AFM height map of a multi-walled CNT. (**d**) Comparison between AFM (i) and TERS (ii) profiles crossing the CNT along the blue line drawn in (**c**). Adapted from [141].

The tips were prepared starting from commercial Si AFM tips (ArrowTM-NanoWorld and TESPA—Bruker), with radii of curvature of about 10 nm and 7 nm, respectively. The procedure is based on the following steps as sketched in Figure 8a [141].

- Oxidation of the Si AFM probe. This is necessary to modify the refractive index from the value of silicon (*n* = 4.4) to that of SiO_2_ (*n* = 1.5) in order to match better the refractive index of the air and tune the plasmon resonance in the visible region. Oxidation is obtained by heating the tips in a muffle furnace at 1000 °C for 10 h to produce a 200 nm SiO_2_ thick layer.
- Coating of the AFM tip using magnetron sputtering. This is done to improve the adhesion of the Ag layer on the tip. First, a bi-layer of Cr-Au (with respective thicknesses of 3 nm and 10 nm) is deposited, and then the layer of Ag is deposited with an optimized thickness of 30 nm.
- Plasma treatment in air. The AFM tip is exposed to the plasma of a radio-frequency discharge (inductively coupling plasma, ICP) sustained by synthetic air. The optimal exposure around 90 s was chosen following a procedure described in [141]. After this treatment, a structured Ag film covered by a Ag oxide (AgO and Ag_2_O [142]) layer is formed.
- Plasma treatment in argon. Further exposure to the Ar-plasma is necessary to reduce the formed Ag oxide. In this case, the exposure time is optimized by monitoring the presence of the oxide using X-ray diffraction (XRD) until it disappears, but preserving the metallic silver nanostructure.

The enhancement factor (EF) of the produced tips was estimated by using crystal violet (CV) dye as probe molecule. Interestingly, after analyzing 22 tips, 91% of them were found to be TERS-active with an average EF = (4.3 ± 3.2) × 10^7^, confirming the high reproducibility of the preparation method. Also in this case, a direct estimation of the spatial resolution was performed by scanning the carbon nanotubes (CNTs) deposited on a glass coverslip. Figure 8c shows the AFM topography image of a multi-walled CNT obtained by scanning a 75 × 75 nm^2^ region. As shown in Figure 8d, the AFM cross-sectional line cut across the CNT exhibits a full width at half maximum (FWHM) of ~30 nm, a value which corresponds to the convolution of the CNT size and the tip radius (~10 nm). Interestingly, the TERS signal across the same line reveals a FWHM of ~10 nm, providing an upper limit of the TERS resolution for the employed tip. Moreover, it is worth mentioning that the Argon-based plasma treatment used to reduce Ag tips oxidized with air-based plasma treatment can be also applied to regenerate Ag-based TERS tips oxidized or contaminated due to exposure to ambient air (for details see ref. [141]).

As mentioned, the SSD-based technique has also been successfully applied to the fabrication of SERS substrates [143]. Here, we mention our recent work aimed at studying the outer part of nanoparticles covered with hyaluronic acid for use in drug delivery [144].

### 6.2. SERS-Active Sensors for the Detection of Pollutant Molecules

Since the SERS technique has now proven to be a powerful analytical technique, it constitutes the core of many sensors for the detection of contaminants in various fields, such as agriculture, and food safety, and even for monitoring pollutant species present both in water and in air [145]. Among the polluting species most present in urban centers, polycyclic aromatic hydrocarbons (PAHs) are certainly the most dangerous due to their proven role in the onset of tumors. Nevertheless, their direct detection by SERS is challenging because of the low chemical affinity with metal surfaces, such as thiols, amines, or carboxylic acids. Several approaches reported in the literature propose to functionalize SERS substrates to promote the interaction between pollutants and the plasmonic sensing platform. However, these strategies present two important limitations: the first is that of adding an intermediate step to the preparation of the substrates, and the second concerns the role of these chemical ligands which eventually interfere with the already crowded Raman spectra.

Herein, we demonstrate the feasibility of label-free detection of pyrene in water by using our highly porous 3D-SERS substrate produced via SSD in plasma [146].

**Figure 9:**
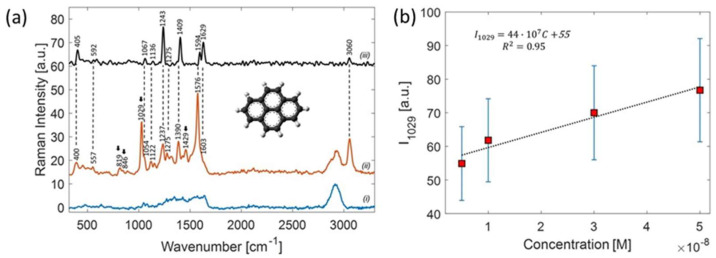
(**a**) Raman spectra of solid pyrene (iii), SERS spectrum (ii) acquired at a concentration of 1 µM pyrene, and, for comparison, (i) SERS spectrum of distilled water evaporated on a SERS substrate. (**b**) Linear trend of band intensity at 1029 cm^−1^ in the lower concentration range with the relative best-fit line. Adapted from [146].

Pyrene is a PAH consisting of four fused benzene rings (C_16_H_10_), resulting in a flat aromatic system. Since pyrene has a low solubility in water (0.135 mg/L at 25 °C), powder samples were preliminary dissolved in methanol (1 µM) and afterwards diluted in Milli-Q water at concentrations ranging from 0.5 µM to 5 nM. Droplets of 20 µL of pyrene solution at the investigated concentrations were dripped into small wells created with a parafilm layer adhered onto the SERS substrate, and allowed to dry at room temperature in a controlled nitrogen atmosphere. From the geometry of the wells, we could easily estimate the molecular surface density σ_mol_ ([C] = 1 mM corresponds to σ_mol_ ~ 10^6^ mol/µm^2^). Figure 9 shows the SERS spectrum acquired using P_laser_ = 180 µW and an integration time of t = 1 s and, for comparison, the Raman spectrum of solid pyrene. As can be noted, with respect to the spontaneous Raman spectrum of solid pyrene (Figure 9a trace (i)), some further peaks (indicated by arrows) appear in the SERS spectrum.

This discrepancy between spontaneous and SERS spectra is quite common and ascribable to the amplification mechanism sensitive to the orientation of vibrational modes with respect the SERS substrate and to the coupling between the hot-spots and the external excitation [147]. Another peculiarity observed in the SERS spectrum of Figure 9a concerns the bands lying in the 2500–4000 cm^−1^ range, which are still amplified despite the large shift with respect to the probe excitation (532 nm), ascribable to the broad plasmon resonance of our SERS substrate.

We found that the porosity promotes the in-filtering of a PAH solution in the interstices and, thus, the “trapping” of the PAH molecules that are eventually adsorbed on the metal surface through non-covalent interactions.

To estimate the limit-of-detection of our method, we performed SERS measurements on pyrene samples with varying concentrations in the range 5–1000 nM. To improve the accuracy, for each concentration, we acquired about 100 spectra at random points on the substrate, and calculated the mean and standard error. Figure 9b shows the concentration-dependent SERS intensity relative to the peaks at 1029 cm^−1^ in the narrow range of concentration 5–50 nM, where we found a linear relationship.

## 7. Conclusions

In this review, we discussed two methods recently developed by our group for the fabrication of plasmonic metallic nanostructures. Both methods have proven to be remarkably interesting both for fabricating planar substrates for SERS spectroscopy and for coating commercial AFM tips with nano-textures for TERS spectroscopy.

The two methods have in common a certain ease of implementation, high efficiency, and spatial reproducibility. However, significant differences emerge between the two methods.

Nanofabrication based on the self-assembling of block copolymers has the advantage of a certain flexibility but has proven to be time-consuming due to the chemical procedure required, and not very reproducible to produce TERS tips because it is linked to a method (dip-coating) which depends on factors that are difficult to control.

On the other hand, the second method developed, based on the solid-state dewetting technique, proved to be more complete, characterized by very fast manufacturing times (a few tens of minutes), and creating reproducible nanostructured silver films both on flat surfaces and on AFM tips. Furthermore, the highly porous (coral-like) structure showed a high density of hot-spots and, therefore, high enhancement factors. Finally, one of the most interesting aspects concerns the problem of aging to which all metallic nanostructures are subject, especially those in silver (which are currently also the most efficient). In fact, the Ar-plasma treatment, which lasts a few tens of seconds, allows the easy regeneration of surfaces or TERS tips subjected to oxidation processes or contamination by spurious molecules adsorbed from the surrounding environment.

Both the SERS substrates and the TERS tips developed by us have been subjected to tests to characterize their performance, and have been successfully used in various experiments for the study of bio-membranes and for the detection of complex molecules of environmental interest.

In conclusion, we expect that an SSD-based protocol, especially dedicated to the fabrication of TERS tips, can contribute to the diffusion of TERS analysis in a broad community of scientists, as a user-friendly analytic tool.

## Data Availability

Data sharing not applicable.
